# *Leptus alberti* n. sp. (Trombidiformes: Erythraeidae) parasitizing free-living colonies of *Apis mellifera*, *Partamona peckolti*, *Paratrigona eutaeniata* and *Tetragonisca angustula* in Totoró and Valle del Cauca, Colombia

**DOI:** 10.1371/journal.pone.0311409

**Published:** 2024-12-16

**Authors:** Brayan Alexander Sanchez-Quilindo, Harby Leandro Pizo-Barona, Javier Antonio Benavides Montaño

**Affiliations:** Department of Animal Science, Universidad Nacional de Colombia, Sede Palmira, Valle del Cauca, Colombia; Norbert Wiener University, PERU

## Abstract

This study examined the ectoparasites in free-living wild bee colonies in Totoró, Cauca Department, and Valle del Cauca, Colombia. Researchers collected 2116 bee specimens: 620 from Cauca (200 *Apis mellifera*, 320 *Partamona peckolti*, and 100 *Paratrigona eutaeniata*) and 1496 from Valle del Cauca (Dagua, Tocota, Buga, Cali, Pradera), including 1498 *A*. *mellifera* and 48 *Tetragonisca angustula*. Using microscopic and stereoscopic techniques and a special taxonomic key, the study identified the first recorded presence of *Leptus (Leptus) alberti* n. sp. (Acari: Erythraeidae) in colonies of free-living Africanized *A*. *mellifera* and stingless bees, including *P*. *peckolti*, *P*. *eutaeniata*, and *T*. *angustula* in Cauca and Valle del Cauca. This finding updates the reported species for South America. The presence of *L*. *(L*.*) alberti* n. sp. was identified in four sample points in the Totoró-Cauca area, with a 75% parasitic prevalence in free-living bee colonies at three of the four localities, specifically in hives located in the wild forest of Totoró. Two Meliponini species were also identified: one in *P*. *peckolti* and another in *P*. *eutaeniata*. In Cauca, the prevalence was 5% for *A*. *mellifera* and 0.3% for Meliponini. In Valle del Cauca, *L*. *alberti* n. sp. was observed in five out of 22 localities, resulting in a 23% prevalence in wild *A*. *mellifera*. Additionally, a 4.16% prevalence (2/48) of *L*. *alberti* n. sp. was found in *T*. *angustula*. Six *A*. *mellifera* specimens tested positive for *L*. *(L*.*) alberti* n. sp., as did one *P*. *peckolti* and one *P*. *eutaeniata* specimen. No other external mites were detected in the collected samples.

## 1 Introduction

Both *A*. *mellifera* and the Meliponini tribe are permanently exposed to a broad range of parasites and pathogens, including metazoans (nematodes, mites), microbes (viruses, bacteria, protozoans, fungi) and parasitoids [1]. Parasites and pathogens are the major source of stress in free-living and managed bee colonies, causing a series of detrimental effects: physiological damage (pathological lesions), metabolic disruptions, imbalances and impaired physiological functions. They also compromise immunological system, weakening their defense mechanisms and reducing their population [2].

*A*. *mellifera* is a honeybee with more than 31 subspecies that was introduced to Latin America in the 17^th^ century, from Africa, Europe, and Western Asia. *A*. *mellifera* scutellate, a hybrid, was subsequently introduced in 1978 and 1997 from Venezuela. Today, 13 identified linages of the African bee haplotypes are present in Colombia territory [3–5]. While frequently described as effective pollinators, recent studies have shown that *A*. *mellifera* may be less efficient when quantified. Furthermore, the honeybee traditional model displaced native bees and decrease pollination of native wildflowers competing with native floral resources [6,7].

Free-living colonies exist in the wild without direct human involvement. They are essentially self-sufficient, depending on natural resources for sustenance and habitat. In the context of free-living colonies, they can consist of a variety of species, such as wild honeybees and formerly managed honeybees that have become free-living. These categories includes colonies of Africanized *A*. *mellifera* (honeybees) and various species of stingless bees.[8]. Since it is challenging to determine whether bees are genuinely wild, descended from colonies that never occupied a beehive, or if they come from free-living colonies that swarmed from a nearby apiary; these bees are denominated as ’free-living’ [8,9].

Colombia is home to over 600 bee species, both in natural and agricultural systems [10]. Include *A*. *mellifera* and stingless bees of the Meliponini tribe (Apidae). There are currently 120 documented stingless bee species in country, including 44 in 175 active meliponaries as of 2020 [11]. The Valle del Cauca and Cauca have 71 species, 18 genera; among them, we can find *Nannotrigona mellaria* [12–14], *P*. *peckolti*, and *T*. *angustula* [13], which had not been previously reported in this Department. Nevertheless, *Paratrigona eutaeniata* has been identified in the Departments of Cundinamarca, Boyacá, Santander, but not in Cauca and Valle del Cauca [15].

*A*. *mellifera* in Colombia faces threats, such as *Varroa destructor*, *Aethina tumida* Murray (Coleoptera: Nitidulidae), *Acaropis woodi* (1.2% prevalence) and *Nosema* spp., (5.1% prevalence) [5,16,17] as well as several viruses [18]. There are few studies on free-living colonies of *A*. *mellifera* or stingless bees; nonetheless, important advances have been implemented in Brazil through the identification of seven novel eukaryotic viruses in high abundance in unhealthy *M*. *quadrifasciata*. [19].

Recently, *Leptus* spp in a Meliponini tribe in Argentina [20]. Mites of the genus *L*. *alberti* n.sp can transmit bacteria, to their host while feeding, which can induce death [21]. Due to the globalization of beekeeping and the importation of genetic material, such as *A*. *mellifera* specimens and other bees, mites have appeared in endemic bees, negatively affecting bee biodiversity in Colombia [22]. It is important to be aware that any new mite-bee association, under suitable conditions, can become a threat to these bees [23]. The objective of this study was to determine the presence of parasitic mites in wild bee populations in the municipality of Totoró (Cauca) and other localities of the Valle del Cauca. This can help to evaluate the potential threat that new parasites may pose to the beekeeping community in the region.

## 2 Materials and methods

### 2.1 Ethics statement

This does not involve participation of specimens’ tissues, vertebrate animals or embryos. Approval for the study was granted by the Ethical Committee Board of the Faculty of Universidad Nacional de Colombia Palmira, letter number 20689, during a meeting held on April 20, 2023. The study falls under the project titled "Interdisciplinary Strategic Alliance aimed at investigating the diversity of mites and parasitic agents associated with Hymenoptera Apidae and devising strategies for their conservation and sustainability in agricultural and livestock production areas in the Pacific region of Colombia." Community clarification sessions were carried out in Cauca and Valle del Cauca to elucidate the project’s objectives and study protocol. Prior to sample collection, consent was obtained from the communities. Additionally, written consent was from the owners before collecting bee samples.

### 2.2 Description area

**Fig 1:**
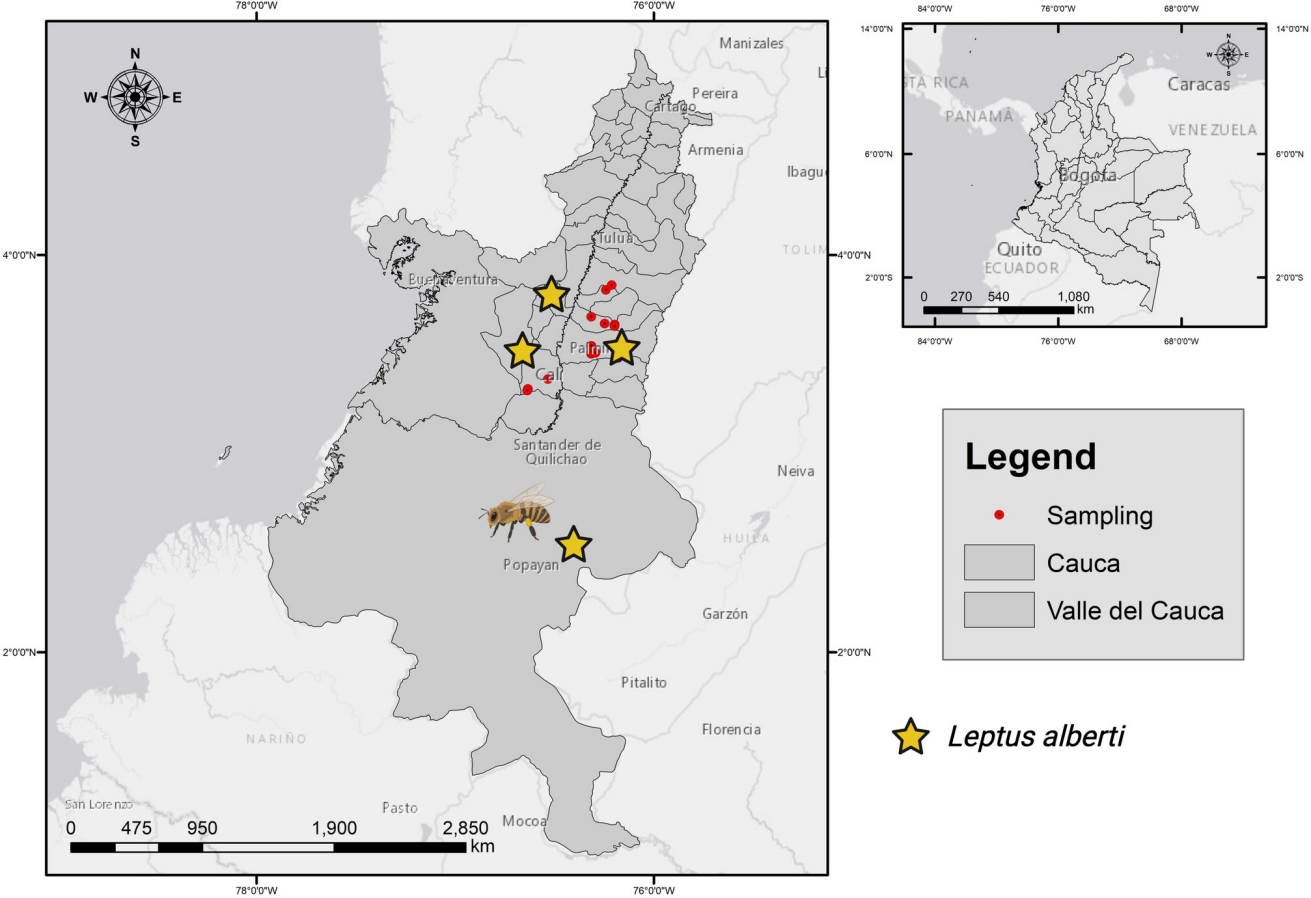
The geographic coordinates. It illustrates the location of agricultural estates within the municipal boundaries of Totoró, ranging in elevation from 2750 to 2500 m.a.s.l. and distributed across the Departments of Cauca and Valle del Cauca. The mapping was generated using Geographic Information Systems Laboratory (GIS) National University of Colombia Palmira headquarters. (2024) and Created with BioRender.com.

**Fig 2:**
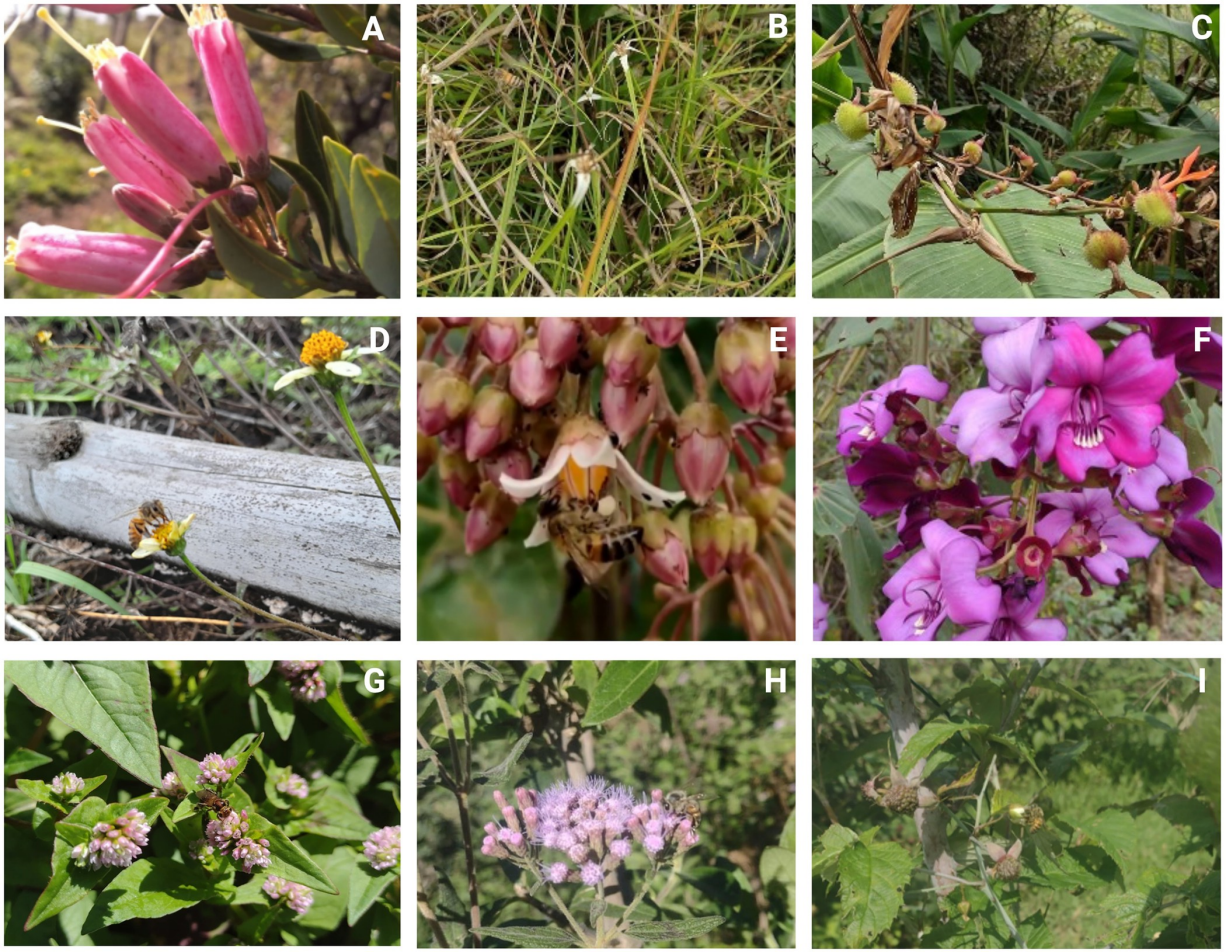
Flora in the surveyed areas. Angucho (*Bejaria resinosa*) (A); White point grass (*Rhynchospora colorata*) (B); Achira (*Canna indica*) (C); Papunga (*Bidens pilosa*) (D), Tree tomato crops (*Solanum betaceum*) (E); Floral tree (*Meriania nobilis*) (F); Zucchini (*Cucurbita pepo*) (G); purple matico (*Aristeguietia lamiifolia*) (H); and Mora castilla (Rubus glaucus) (I). Created with BioRender.com.

Totoró is a region in Colombia characterized by significant biological and cultural diversity, with temperatures ranging from 0°C to 22°C and a broad range of altitudinal zones, from 2200 to 3800 meters above sea level (Fig 1). Indigenous communities include the Nasas, Polindaras, and Tontotuna [24]. The local inhabitants are primarily engaged in agricultural activities, which involve the utilization of trees for various purposes such as timber, ornamental use, water protection, fruit cultivation, and erosion control. Furthermore, their livelihoods revolve around managing their own seeds in gardens, species selection, planting, exchange, and sale. Additionally, animal husbandry is an integral part of their activities [25]. The Piedra Grande farm, situated in Miraflores village, served as the initial sampling location and spans across 8 hectares, 2 are specifically allocated to the cultivation of Castilla blackberry (*Rubus glaucus*), tomato (*Solanum betaceum*), and zucchini (*Cucurbita pepo*) (Fig 2). Remaining 6 hectares are designated for cattle husbandry and stewardship practices. The wild hive was identified at an approximate distance of 300 meters from the farm entrance, encompassed by a biodiverse array of indigenous plant species (Fig 2). The El Recuerdo farm, situated in the La Palma hamlet, is located at a 45-minute walking distance from the Piedra Grande farm. This agricultural establishment is dedicated to the intensive rearing of Cebuina breed cattle. In addition, the farm features a limited representation of indigenous flora, incorporating specific shrub species and notably showcasing the flourishing of white point grass (Fig 2). During the sampling conducted within Valle del Cauca, positive observations were made regarding the presence of the ectoparasite *Leptus* spp. The assessments were specifically carried out within apicultural settings, focusing on wild *A*. *mellifera* colonies located in Dagua-Tocota, Buga, Pance, Pradera, and the Topacio reserve in Cali. The final sample involved a natural hive hosting *Tetragonica angustula*.

### 2.3 Sampling points

The field study focused on six diverse areas within the Departments of Cauca and Valle del Cauca. In the Cauca Department, mites (Trombidiformes: Erythraeidae) were found in the Municipality of Totoró (Lat: 2°30′37″N, Long: 76°24′07″W, Alt: 2750, 14°C, Biological Register **ID: H.01**.), two sampling point in Miraflores village (Lat: N 2°33′25″, Long: 76°25′21″ W, Alt: 2700 m.a.s.l., 19°C); one sampling in La Palma-Totoró (Lat: 3°32′34″ N, Long: 76°26′26″W, alt: 2481 m.a.s.l., 14°C, **ID:H.04**); and the last one in Bella Vista-Totoró (Lat: 2°34’36.6" N, longitude: 76°27’20.4" W, Alt: 2500 m.a.s.l., 14°C, **ID:H.25**). Fig 1 illustrates the sampling locations: in the Valle del Cauca Department, in Dagua-Tocota (Lat: 3°30’28" N, Long: 76°38’55" W, Alt:1520 m.a.s.l. 17°C, **ID: H.02**.**)**; the Agroecological farm **“**El Porvenir-Buga” (Lat: 3°50’55.0"N, Long: 76°12’46.0"W, Alt: 2481 m.a.s.l., 19°C **ID: H.014**.**);** Natural forest reserve, Topacio. Cali, located at (Lat: 3°19’03.6"N, Long: 76°38’15.2"W, Alt: 2481 m.a.s.l., 14°C **ID: H.017**.); High Mountain of Pueblito Pance (El Trueno). (Lat: 3°19’03.6"N, Long: -76°38’15.2"W, Alt: 2481 m.a.s.l., 18°C **ID: H.018**); Villa El Mesón. Pradera (Lat: 3°29’55.9"N, Long: 76°11’23.3"W, Alt: 1869 m.a.s.l., 20°C **ID: H.019**).

### 2.4 Study and sample size

The study was conducted from February 2023 to February 2024. In order to calculate the sample size, we used the formula n = E_2_Z_2_⋅p⋅(1−p)/E^2^, where n = sample size Z = Z-score corresponds to the desired confidence level (for 95% confidence, Z ≈ 1.96), p = estimated proportion of the Meliponini species population that is unknown in the area of interest. We used 0.5% for maximum variability with margin of error E. $n=\frac{1.962\cdot 0.5\cdot (1-0.5)}{0.5^{2}},\;n=\frac{3.8416\cdot 0.25}{0.0025},\;n=\frac{0.9604}{0.0025},\;n=384$. This study adopted an observational approach, closely examining exposure to parasites within each sample. A representative sample from each location underwent scrutiny at a specific moment and place [26]. We collected a total of 2116 individuals from 25 located points to get enough specimens for analysis. The data was analyzed measuring the prevalence of the disease, and it was calculated (No. of specimens parasitized/Total no. of specimens examined) x 100.

**Fig 3:**
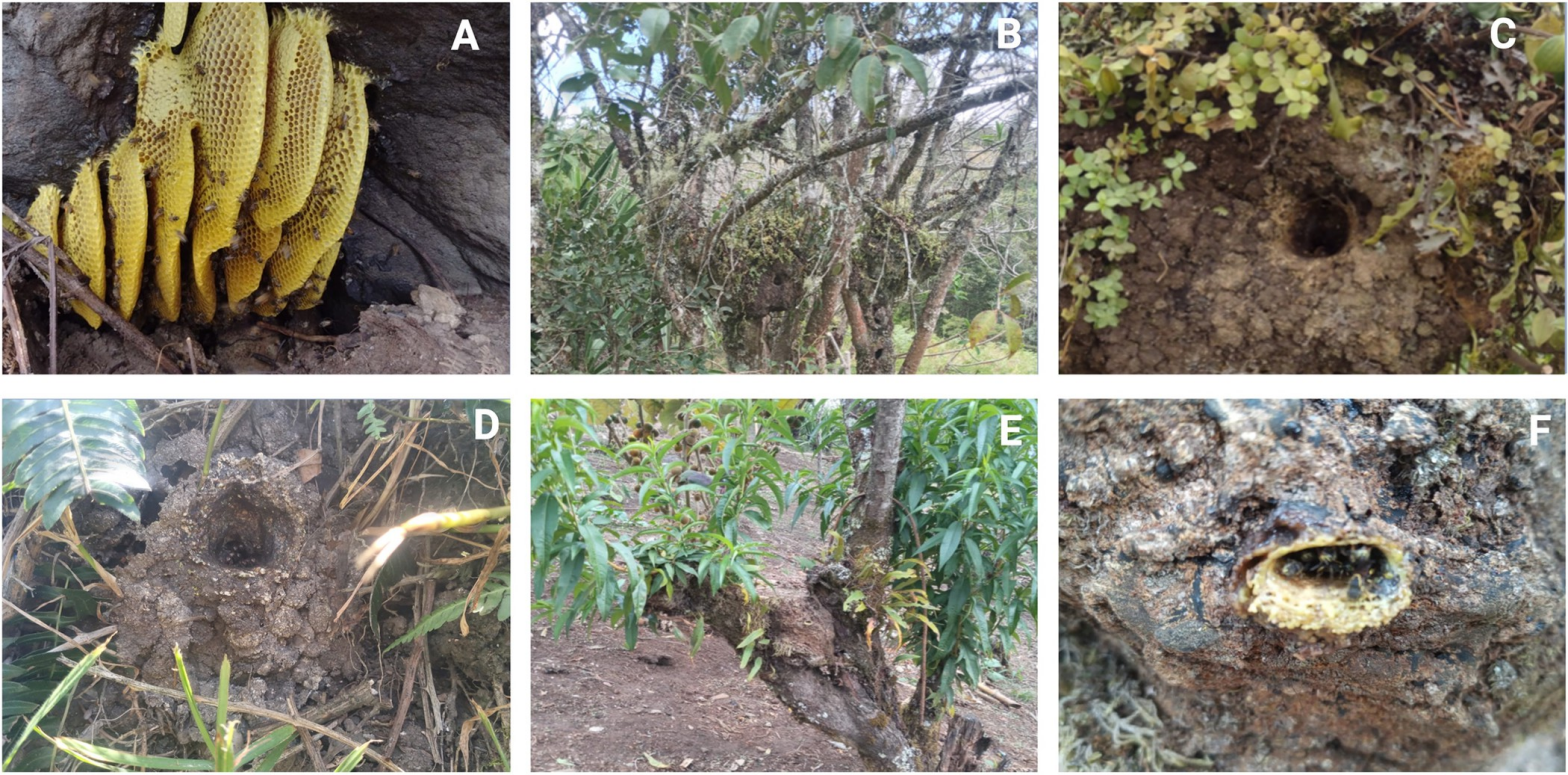
Farm Piedra Grande. Habitats of wild bees (Apini) in Miraflores–Totoró. (A); Positioning of *P*. *peckolti* Meliponini wild bees on a beach milk tree, *P*. *(Euphorbia laurifolia])* (B); Nest of wild bee specimens (Meliponini) in La Palma (Finca El Recuerdo) (C); Nest of *P*. *peckolti* wild bees (Meliponini) in Miraflores–Totoró, (D); Location of a wild hive on a peach tree, *Prunus persica*, *P*. *peckolti* (E); Nest of wild bee specimens *P*. *eutaeniata* (Meliponini) in Miraflores, Bellavista village, (F), *P*. *eutaeniata* over *Persicaria nepalensis* (G), Mantico morado, *Ipomea purpurea* (I), *Rubus glaucus* (H). Created with BioRender.com.

To detect ectoparasites, including *Leptus* spp, *Varroa* spp, *A*. *woodi*, among other mites, a comprehensive analysis was conducted in the Departments of Cauca and Valle del Cauca. In the first region, a total of 620 individuals from wild hives were processed: 200 *A*. *mellifera* and 420 stingless bees. Specifically, 115 *P*. *peckolti* were examined in Miraflores-Totoró, 205 in La Palma-Totoró, and 100 *P*. *eutaeniata*in in Bellavista-Totoró (Fig 3). In the second Department, the investigation entailed the examination of a total of 1496 individuals derived from both apiaries and wild hives. This cohort comprised 1448 *A*. *mellifera*, with the following spatial distribution: 830 specimens in Dagua-Tocota, 366 in the agroecological farm “El Porvenir” located in Buga municipality, 91 samples collected in the High Mountain of Pueblito Pance (El Trueno), and 161 free-living colonies of *A*. *mellifera* in Villa El Mesón, Pradera. Additionally, 48 individuals of wild *T*. *angustula* underwent scrutiny in Reserva Topacio (Fig 3).

### 2.5 Mounting, microscopy, and image analysis

The bees were captured using a wide-mouthed jar containing 70% alcohol. Parasitic larvae of the Trombidiformes: Erythraeidae family were collected from *A*. *mellifera*, *P*. *peckolti*, *P*. *eutaeniata* and *T*. *angustula* in Totoró, Cauca, and Valle del Cauca, Pacific of Colombia. The collection was deposited in CEUNP-70 at the Universidad Nacional de Colombia, Palmira campus. The alcohol fixed specimens were slide-mounted in Hoyer’s medium. They were observed using a Leica S8APO stereoscope and both ZEISS PrimoVert and Primo Star AxioCam ICc microscopes. The mites were examined using two microscopy techniques: Phase-contrast microscopy (PCM) and Light microscopy (LM). Taxonomic keys were used for precise and rigorous identification [27–32]. Minimum and maximum values of the morphometric measurements were registered during captures of individuals for which photogrammetric data existed. 11 specimens were studied: H01.P01.I017-SL1, H01.P01.I81-SL1, H01.P01.I093-SL1, H01.P01.I116-SL1, H01,P01.I189-SL1, H01,P01.I189-SL2, H04.P02.I178-SL1, H04.P02.I178-SL2(r), H25.P01.I018-SL1, H18.P02.I036-SL1, and H19.P02.I015-SL1. All measurements are given in micrometers, with the range followed by the mean. The terminology and measurements generally follow Southcott (1992) [27], Haitlinger (2016) [30], Saboori, (2020) [31], and Haitlinger (1991) [32]. This evaluation was carried out in the Parasitology, Immunology, and Infectious Diseases Laboratory (PARINEI), located at the Lab Farm Mario González Aranda, Experimental Center of the Universidad Nacional de Colombia, Palmira Campus, Valle del Cauca.

### 2.6 Statistical analysis

The statistical analysis was conducted using GraphPad Prism 10 software. Comparisons of samples collected in Cauca and Valle del Cauca for *Leptus alberti* n. sp. were performed using a one-way analysis of variance (ANOVA), followed by Bonferroni’s multiple comparison post hoc test.

## 3 Results

**Fig 4:**
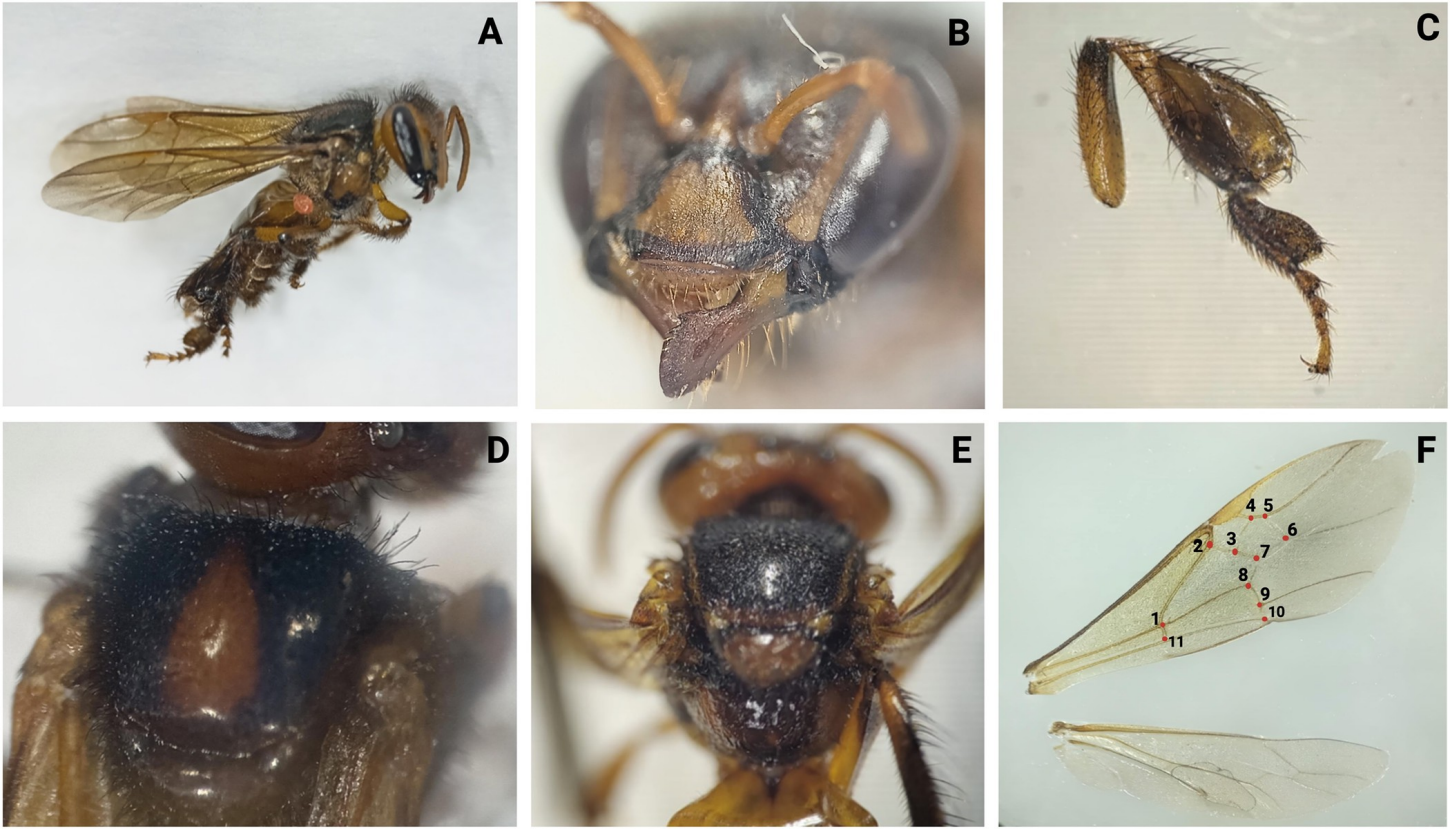
*P*. *peckolti* (H.04.P02.I178). Infestation by *Leptus (L) alberti*.*n*. *spp*, characterized by a black tegument with brown coloring. The hind legs femur displays a mite attached to the trochanter (A). *P*. *peckolti* features a malar mouth with a small lateral tooth and spoon-shapedarea (B), spoon-shaped corbiculae (C), scutellum without a medial notch (D), and a propodeum larger, measuring 1–1.5 times the scutellum’s length; notable hairs present on both ventral and dorsal thoracic areas (E). The wings exhibit a reddish membrane with a pentagon (3-4-5-6-7) (F), with overall dimensions ranging between 5.5 and 6.3 mm [15]. Created with BioRender.com.

**Fig 5:**
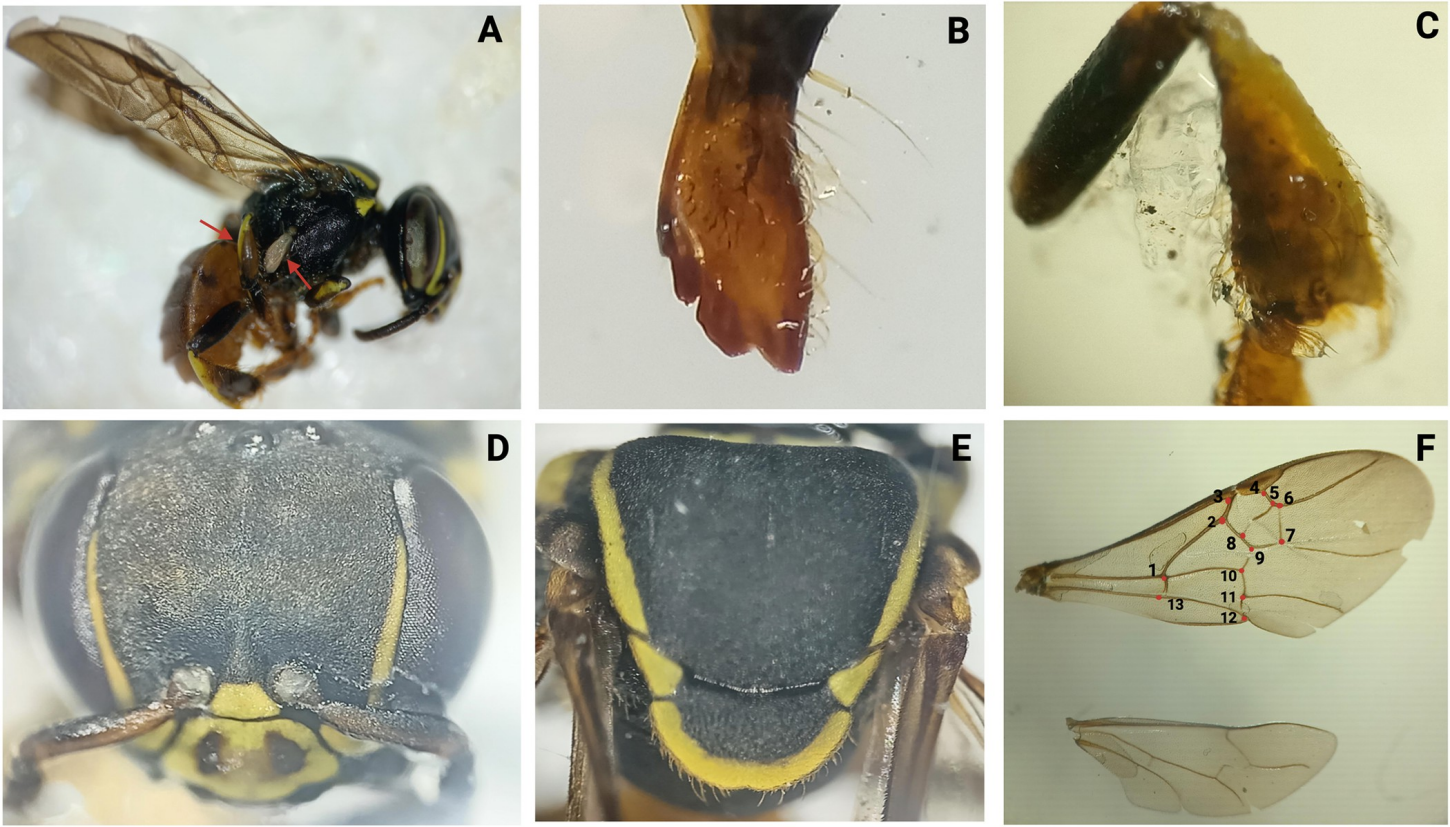
*Paratrigona eutaeniata*. H.25.P01.I018. Mite attached to the lateral thorax (A); malar with four denticles (B); tibia in a triangular shape (C); ocellus and exposed alveolar areas; yellow clypei and two yellow stains in the shape of an inverse comma, at the lateral orbicular margin from medium ocellus (D); lack of medial notch (E); hexagon-shaped wings (F) [15]. Created with BioRender.com.

We identified the presence of *L*. (L) *alberti* n. spp. in four sample points in the area of Totoró-Cauca, within the Department of Cauca (Table 1, see also S1 Fig). In 3 of the 4 localities, parasitic prevalence was 75% in free-living colonies of bee hives. Specifically, three hives located in the wild forest within the municipality of Totoró. We also identified two Meliponini species: one in *P*. *peckolti* (Fig 4) and another in *P*. *eutaeniata* (Fig 5). The total population, as previously described, included 200 *A*. *mellifera*, 320 *P*. *peckolti*, and 100 *P*. *eutaeniata*.

**Table 1:** Taxonomical identification key for the larvae stages of *L*. *alberti* n. sp., parasitizing free-living colonies of *A*. *mellifera*, *P*. *peckolti*, *P*. *eutaeniata*and *T*. *angustula* in Totoró–Cauca, and Valle del Cauca–Pacific of Colombia. We employed taxonomic keys in the Neotropical regions [27,30–32].

**1**	Scutum with two or more additional scutalae, placed beyond setae PL (subgenus *Amaroptus*). . . .. . . .. . . .. . . .. . . .. . . .. . . .. . . .. . . .. . . .. . . .. . . .. . . .. . . .. . . .. . . .. . . .. . . .. . . .. . . .. . . .. . . .. . . .. . . .. . . .. . . .. . . .. . . . *L*. (A.) vuki (Haitlinger, 2000) [40]; Peru	**No**
	- Scutum without such setae (subgenus *Leptus*). . . .. . . .. . . .. . . .. . . .. . . .. . . .. . . .. . . .. . . .. . . .. . . .. . . .. . . .. . . .. . . .. . . .. . .. . ..2 **(Fig 5A)**	**Yes**
**2**	Palpgenu and palpfemur with two setae each. . . .. . . .. . . .. . .. . .*L*. (L.) maldonadoicus (Haitlinger, 2000) [40]; Peru	**No**
	Palpgenu with one or two setae, palpfemur with only one seta. . . .. . . .. . . .. . . .. . . .. . . .. . . .. . . .. . . .. . . .. . . .. . . .. . . . 3 **(Fig 5B)**	**Yes**
**3**	Between coxae I four sternalae, between coxae II six sternalae and between coxae II- III more than four setae. . . .. . . .. . . .. . . .. . . .. . . .. . . .. . . .. . . .. . . .. . . .. . . .. . . .. . . .. . . .. . . .. . . . *L*. (L.) ursyni (Haitlinger, 1991) [32]; Chile	**No**
	- Between coxae I and II two sternalae, between coxae II-III not more than four setae. . . .. . .. . ..4 **(Fig 5C)**	**Yes**
**4**	Palpegenu with two setae. . . .. . . .. . . .. . . .. . . .. . . .. . . .. . . .. . . .. . . .. . . .. . . .. . . .. . . .. . . .. . . .. . . .. . . .. . . .. . . .. . . .. . . .. . . .. . . .. . . .. . . .. . . .. . . .. . . .. . . .. . . .. . . ..5	**No**
	Palpgenu with one seta ……………………………………………………………………………..8 **(Fig 5B)**	**Yes**
**5**	Posterolateral scutal seta (PL) off scutum. . . .. . . .. . . .. . . .. . . .. . . .. . . .. . . .. . . .. . . .. . . .. . . .. . . .. . . .. . . .. . . .. . . .. . . .. . . .. ………. . . .. . . .. . . .. . . .. . . .. . . .. . . .. . . .. . . .. . . .. . . .. . . .. . . .. . . .. . . .. . . .. . . .. . . .. . . .. . . .. . . .. . . .. . . .. . . .. *L*. (L.) *lomani* (Oudemans, 1902) [50]; Chile	
	Setae PL on scutum. . . .. . . .. . . .. . . .. . . .. . . .. . . .. . . .. . . .. . . .. . . .. . . .. . . .. . . .. . . .. . . .. . . .. . . .. . . .. . . .. . . .. . . .. . . .. . . .. . . .. . . .. . . .. . . .. . . .. . . .. . . . 6 **(Fig 5A)**	
**6**	L > 100, W > 120.* *.* *.* *..* *.* *.* *..* *.* *.* *..* *.* *.* *..* *.* *.* *..* *.* *.* *..* *.* *.* *..* *.* *.* *..* *.* *.* *..* *.* *.* *..* *.* *.* *..* *.* *.* *..* *.* *.* *..* *.* *.* *..* *.* *.* *..* *.* *.* *..* *.* *.* *..* *.* *.* *..* *.* *..* *.* *.. *L*. (L.) *hringuri* (Haitlinger, 2000) [40]; Peru	
	L <100, W <100. . . .. . . .. . . .. . . .. . . .. . . .. . . .. . . .. . . .. . . .. . . .. . . .. . . .. . . .. . . .. . . .. . . .. . . .. . . .. . . .. . . .. . . .. . . .. . . .. . . .. . . .. . . .. . . .. . . .. . . .. . . .. . . .. . . .. . . .. . . .. . .. . .. 7	
**7**	fD 60, W < 78, Ti III < 135 …… L (L) cabareticus (Haitlinger, 2004) [39]; Dominican Republic, Guadeloupe	
	fD 82, W > 80, Ti III >. . . .. . . .. . . .. . . .. . . .. . . .. . . .. . . .. . . .. . . .. . . .. . . .. . . .. . . .. . .. . .. *L*. (L.) iguacuicus (Haitlinger, 2004) [39]; Brazil	
**8**	Genu III with 7–9 solenidia… *L*. (L.*)* multisolenidiae (Mayoral & Barranco, 2011) [51]: 2011, French Guiana	**No**
	Genu III with other number of solenidia or without solenidia. . . .. . . .. . . .. . . .. . . .. . . .. . . .. . . .. . . .. . . .. . . .. . .. . .. 9 **(Fig 5E)**	**Yes**
**9**	Genu I with five solenidia. . . .. . . .. . . .. . . .. . . .. . . .. . . .. . . .. . . .. . . .. . . .. . . .. . . .. . . .. . . .. . . .. . . .. . . .. . . .. . . .. . . .. . . .. . . .. . . .. . . .. . . .. . . .. . . .. . . .. . . .. . . .. 10	**No**
	Genu I with one or no solenidion. . . .. . . .. . . .. . . .. . . .. . . .. . . .. . . .. . . .. . . .. . . .. . . .. . . .. . . .. . . .. . . .. . . .. . . .. . . .. . . .. . . .. . . .. . . .. . .. . .. 11 **(Fig 5F)**	**Yes**
**10**	10 Genu II with one solenidion, telofemur I without solenidion. . . .. . . .. . . .. . . .. . . .. . . .. . . .. . . .. . . .. . . .. . . .. . . .. . . .. . . .. . . .. . . .. . . .. . . .. . . .. . . .. . . .. . . .. . . .. . . .. . . .. . . .. . . .. . . .. . . .. . . .. . . .. . . .. . . .. . . .. . . .. . .. . .. *L*. (L.) stieglmayri (Oudemans, 1905); Brazil	
	Genu II without solenidion, telofemur with three solenidia. . . .. . . .. . . .. . . .. . . .. . . .. . . .. . . .. . . .. . . .. . . .. . . ... . . .. . . .. . . .. . . .. . . .. . . .. . . .. . . .. . . .. . . .. . . .. . . .. . . .. . . .. . . .. . . .. . . .. . . .. . . .. . . .. . . .. . . .. . . .. . . .. . . .. . . . *L*. (L.) schedingi (Oudemans, 1911); Chile	
**11**	Tibia III with two solenidion. . . .. . . .. . . .. . . .. . . .. . . .. . . .. . . .. . . .. . . .. . . .. . . .. . . .. . . .. . . .. . . .. . . .. . . .. . . .. . . .. . . .. . . .. . . .. . . .. . . .. . . .. . . .. . . .. . . .. . . .. 12	**No**
	- Tibia III with one solenidion. . . .. . . .. . . .. . . .. . . .. . . .. . . .. . . .. . . .. . . .. . . .. . . .. . . .. . . .. . . .. . . .. . . .. . . .. . . .. . . .. . . .. . . .. . . .. . . .. . .. . . 13 **(Fig 5G)**	**Yes**
**12**	L < 130, W < 120, AL < 70. . . .. . . .. . . .. . . .. . . .. . . .. L. (L.) filipinae (Haitlinger, 2000) [40]; Costa Rica, Belize, Mexico	
	- L > 150, W > 170, AL > 80. . . .. . . .. . . .. . . .. . . .. . . .. . . .. . . .. . . .. . . .. . . .. . . .. . .. . . *L*. (L.) stefani (Haitlinger, 1991) [32]; Colombia	
**13**	13 Genu I without solenidion. . . .. . . .. . . .. . . .. . . .. . . .. . . .. . .. . ..*L*. (L.) gagzoi (Oudemans, 1910) [49]; Panama, Trinidad	**No**
	Genu I with one solenidion. . . .. . . .. . . .. . . .. . . .. . . .. . . .. . . .. . . .. . . .. . . .. . . .. . . .. . . .. . . .. . . .. . . .. . . .. . . .. . . .. . . .. . . .. . . .. . . .. . . .. . . .. . . . 14 **(Fig 5F)**	**Yes**
**14**	Genu II with one solenidion. . . .. . . .. . . .. . . .. . . .. . . .. . . .. . . .. . . .. . . .. . . .. . . .. . . .. . . .. . . .. . . .. . . .. . . .. . . .. . . .. . . .. . . .. . . .. . . .. . . .. . . .. . . .. . . .. . . .. . . . 15	**No**
	- Genu II without solenidia. . . .. . . .. . . .. . . .. . . .. . . .. . . .. . . .. . . .. . . .. . . .. . . .. . . .. . . .. . . .. . . .. . . .. . . .. . . .. . . .. . . .. . . .. . . .. . . .. . . .. . . .. . . .. 16 **(Fig 5I)**	**Yes**
**15**	Two setae between and anterior to coxae III. . . .. . . .. . . .. . . .. . . .. . . .. . . .. . . .. . . .. . . .. . . .. . . .. . . .. . . .. . . .. . . .. . . .. . . ... . . .. . . .. . . .. . . .. . . .. . . .. . . .. . . .. . . .. . . .. . . .. . . .. . . .. . . .. . . .. . . .. . . .. . . .. . . .. . . .. . . .. . . .. . .. . .. *L*. (L.) oudemansi (Karpinnen, 1958); Surinam	
	Four setae between and anterior to coxae III. . . .. . . .. . . .. . . .. . . .. . . .. . . .. . . .. . . .. . . .. . . .. . . .. . . .. . . .. . . .. . . .. . . .. . . ... . . .. . . .. . . .. . . .. . . .. . . .. . . .. . . .. . . .. . . .. . . .. . . .. . . .. . . .. . . .. . . .. . . .. . . .. . . .. . . .. . . .. . . .. . . .. . .. . . *L*. (L.) sieversi (Oudemans, 1911); Venezuela	
**16**	Ti III > 450. . . .. . . .. . . .. . . .. . . .. . . .. . . .. . . .. . . .. . . .. . . .. . . .. . . .. . . .. . . .. . . .. . . .. . . .. . . .. . . .. . . .. . .. . .. *L*. (L.) stolae (Haitlinger, 1987) [53]; Brazil	**No**
	- Ti III < 400. . . .. . . .. . . .. . . .. . . .. . . .. . . .. . . .. . . .. . . .. . . .. . . .. . . .. . . .. . . .. . . .. . . .. . . .. . . .. . . .. . . .. . . .. . . .. . . .. . . .. . . .. . . .. . . .. . . .. . . .. . . .. . . .. . . .. . .. . . 17 **(Fig 5J)**	**Yes**
**17**	Ti III< 190. . . .. . . .. . . .. . . .. . . .. . . .. . . .. . . .. . . .. . . .. . . .. . . .. . . .. . . .. . . .. . . .. . . .. . . .. . . .. . . .. . . .. . . .. . . .. . . .. . . .. . . .. . . .. . . .. . . .. . . .. . . .. . . .. . . .. . . .. . . .. 18 **(Fig 5J)**	**Yes**
	- Ti III > 190. . . .. . . .. . . .. . . .. . . .. . . .. . . .. . . .. . . .. . . .. . . .. . . .. . . .. . . .. . . .. . . .. . . .. . . .. . . .. . . .. . . .. . . .. . . .. . . .. . . .. . . .. . . .. . . .. . . .. . . .. . . .. . . .. . . .. . . .. . . .. . . .. . .. . . 24	**No**
18	AW < 66. . . .. . . .. . . .. . . .. . . .. . . .. . . .. . . .. . . .. . . .. . . .. . . .. . . .. . . .. . . .. . . .. . . .. . . .. . . .. . . . *L*. (L.) *onnae* (Haitlinger, 2000) [40]; Brazil, Mexico	**No**
	- AW > 66. . . .. . . .. . . .. . . .. . . .. . . .. . . .. . . .. . . .. . . .. . . .. . . .. . . .. . . .. . . .. . . .. . . .. . . .. . . .. . . .. . . .. . . .. . . .. . . .. . . .. . . .. . . .. . . .. . . .. . . .. . . .. . . .. . . .. . .. . . 19 **(Fig 5K)**	**Yes**
**19**	AW > 88. . . .. . . .. . . .. . . .. . . .. . . .. . . .. . . .. . . .. . . .. . . .. . . .. . . .. . . .. . . .. . . .. . . .. . . .. . . .. . . .. . . .. . . .. . . .. . . .. . . .. . . .. . . .. . . .. . . .. . . .. . . .. . . .. . . .. . . .. . . . 20 **(Fig 5K)**	**Yes**
	- AW < 88. . . .. . . .. . . .. . . .. . . .. . . .. . . .. . . .. . . .. . . .. . . .. . . .. . . .. . . .. . . .. . . .. . . .. . . .. . . .. . . .. . . .. . . .. . . .. . . .. . . .. . . .. . . .. . . .. . . .. . . .. . . .. . . .. . . .. . . .. . . .. . . .. . . .. . . .. 21	**No**
**20**	PW 86–90, Ti I 94–100, anterior border of scutum deeply concave. . . .. . . .. . . .. . . .. . . .. . . .. . . .. . . .. . .. . ... . . .. . . .. . . .. . . .. . . .. . . .. . . .. . . .. . . .. . . .. . . .. . . .. . . .. . . .. . . .. . . .. . . .. . . .. . . .. . . .. . . .. . . .. . . .. . . .. . . .. . . .. *L*. (L.) *adaminae* (Haitlinger, 2004) [39]; Brazil	**No**
	- PW 120, Ti I 122, anterior border of scutum almost straight. . . .. . . .. . . .. . . .. . . .. . . .. . . .. . . .. . . .. . . .. . . .. . . .. . . .. . . .. . . .. . . .. . . .. . . .. . . .. . . .. . . .. . . .. . . .. . . .. . . .. . . .. . . .. . . .. . . .. . . .. . . .. . . .. . . .. . . .. . . .. . . .. . . .. . . .. . .. . . *L*. (L.) *alberti* (Haitlinger, 1991) [32]; Brazil	**Yes**

In Cauca, a prevalence of 5% was noted for *A*. *mellifera* and 0.3% for Meliponini. In Valle del Cauca, *L*. *alberti* n. spp. was observed in five out of 22 localities, resulting in a prevalence of 23% in wild *A*. *mellifera* (H.02, H14,H18,H19). Notably, a prevalence of 4.16% (2/48) of *L*. *alberti* n. spp. was identified in *T*. *angustula* (codes H.17.P01.I009H, H17.P02.I010). Six *A*. *mellifera* specimens from the collected material tested positive for *Leptus* (L) *alberti* n.sp spp., (SL) (codes H01.P01.I017, H01.P01.I044, H01.P01.I081, H01.P01.I093, H01.P01.I116, H01.P01.I189). Additionally, one *P*. *peckolti* specimen was identified as positive (code H.04.P02.I178), as well as a *P*. *eutaeniata* (code H.25.P01.I018). *Varroa*, *Euvarroa*, and other external mites were not observed in the collected samples.

**Fig 6:**
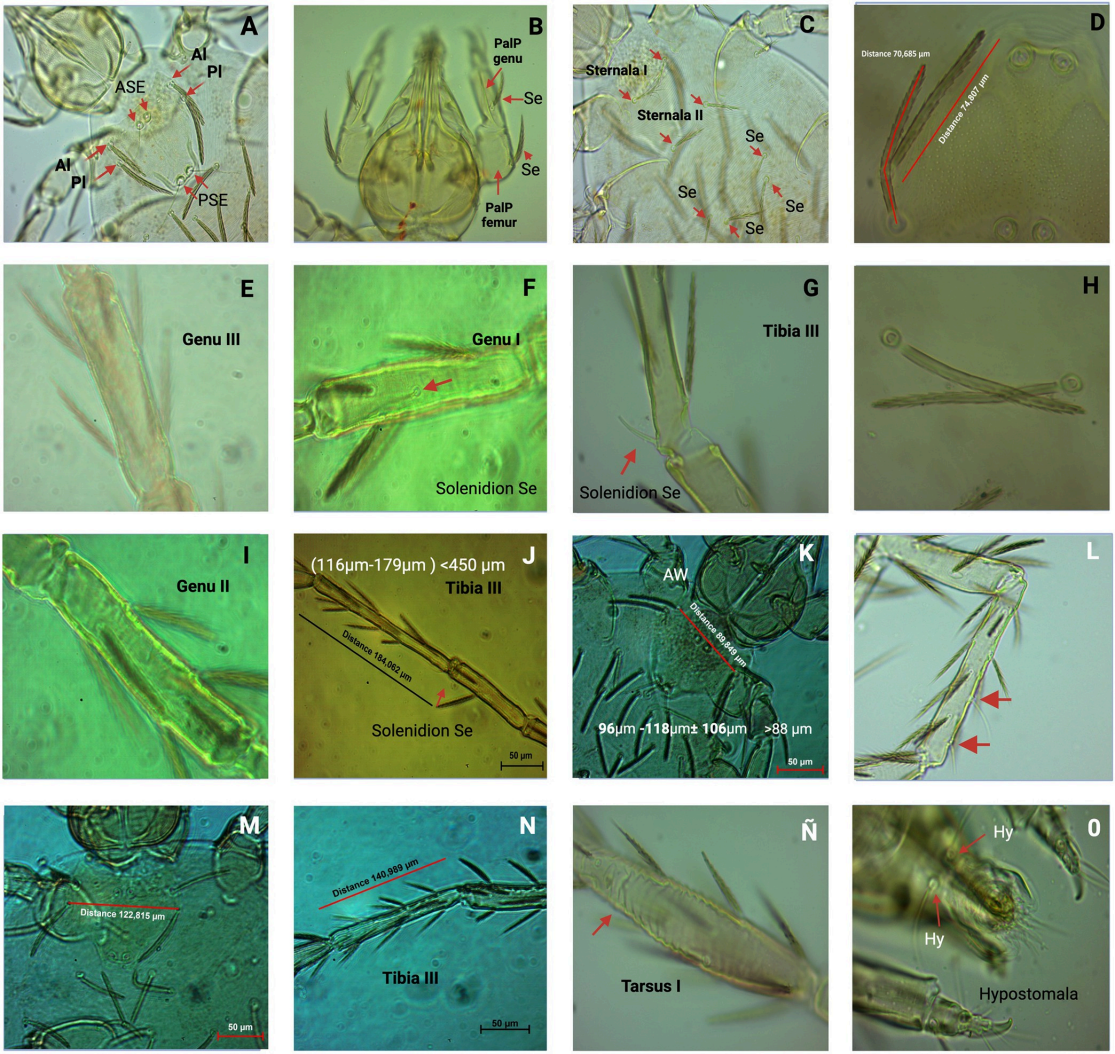
*L*. *alberti* n. spp. Specimens were collected from free-living colonies of *A*. *mellifera* (H.01.P01.I189), *P*. *peckolti* (H.04.P02.I178) and *P*. *eutaeniata* (H.25.P01.I018). The collected material underwent microscopic examination at 20X, 40X, and 100X magnifications: (H.01.P01.I189-SL, H.01.P01.I189-SL2, H.04.P02.I178-SL1, H.25.P01.I018-SL1). Scutum without such setae (subgenus *Leptus*), Setae PL on scutum **(A)**; wide shield without cuticular lines, moderately chitinized, with slight porosity but without striae (stippling), equilateral triangle shape **(A)**; Palp genu with one or two setae, palp femur with only one Se **(B)**, Palp genu with one seta **(B),** Between coxae I and II two sternale, between coxae II-III not more than four setae **(C)**, AL shorter than PL, ciliate (**5D**), Genu III without solenidia **(E)**, Genu I with one solenidion **(F)**, Tibia III with one solenidion **(G),** Dorsum of the idiosome with about 48 ciliated setae **(H)**, Genu II without solenidia **(I),** Ti III < 400 **(J),** Ti III< 190 **(J),** AW > 66 **(K),** AW > 88 **(K),** Circular eyes, 16.47 μm wide, on small oval or pyriform ocular sclerites **(K)**, Tibia I with two specialized setae **(L),** Striated tarsus I (**M**), (**N**), **(Ñ)**, naked hypostomala, with two hypostomal setae **(O)**, PW 120, Ti I 122, anterior border of scutum almost straight (A). Created with BioRender.com.

The specimens utilized for the classification of *Leptus* spp. were obtained from free-living colonies of *A*. *mellifera* in Totoró, Cauca (H01.P01.I017-SL1, H01.P01.I81-SL1, H01.P01.I093-SL1, H01.P01.I116-SL1, H01,P01.I189-SL1; H01,P01.I189-SL2); from *P*. *peckolti* (H04.P02.I178-SL1; H04.P02.I178-SL2.r); from *P*. *eutaeniata* (H25.P01.I018-SL1); from feral wild bees in “El Meson” (Palmira, Valle del Cauca) (H19.P02.I015-SL1); and from the high mountain of Pueblito Pance “El Trueno”, Valle del Cauca (H18.P02.I036-SL1), Tables 1 and 2, (Fig 6). No significant difference among populations of *L*. (L) *alberti* n. spp in Cauca and Valle del Cauca (S1 Fig). *Varroa*, *Euvarroa*, and other external mites were not detected in the collected samples. The mites were systematically identified and cataloged with corresponding codes, as presented in Tables 1 and 2, and (Fig 6).

**Table 2:** Data of larvae of *L*. *alberti* n. sp. Parasitizing feral *A*. *mellifera*, *P*. *peckolti*, *P*. *eutaeniata*and *T*. *angustula* in Totoró, Cauca, and Valle del Cauca—Pacific of Colombia.

Character	H01.P01.I017-SL1	H01.P01.I81-SL1	H01.P01.I093-SL1	H01.P01.I116-SL1	H01,P01.I189-SL1	H01,P01.I189-SL2	H04.P02.I178-SL1	H04.P02.I178-SL2(r)	H25.P01.I018-SL1	H19.P02.I015-SL1	H18.P02.I036-SL1	Range	Mean	s.d
**AW**	105,28	96,511	108,72	91,3	98,71	104,5	118,022	95,87	118,12	104,68	101,396	91–118	103,9	8,6
**PW**	118,75	113,557	124,12	103,72	108,19	117,7	129,2	113,6	131,25	120,8	117,59	103–131	118,0	8,3
**SBp**	13,22	13,452	14,64	15,11	9,98	14,82	13,99	13,72	17,14	13,45	13,042	9–17	13,9	1,7
**PL**	66,2	72,736	75,3	69,9	73,23	67,64	73,55	69,51	73,37	64,28	60,46	60–75	69,7	4,6
**AL**	55,12	54,983	50,11	64,2	62,57	49,8	68,6	56,8	71,91	61,89	59,66	49–72	59,6	7,1
**SBa**	12,18	12,126	15,4	14,36	8,76	11,33	17,14	13,91	17,8	12,27	11,337	8–18	13,3	2,7
**W**	129,74	114,77	136,56	113,4	123,91	129,1	138,89	124,52	142,07	132,46	127	113–142	128,4	9,1
**L**	104,22	110,938	88,36	100,86	105,36	87	107,73	99,6	116,06	89,57	91,112	87–116	100,1	9,9
**ISD**	72,14	75,659	71,41	66,5	72,3	70,53	73,44	69,63	71,19	69,97	67,55	66–75	70,9	2,6
**LX**	24,94	18,94	13,99	20,32	21,77	20,053	18,38	13,59	28,03	22,71	20,14	13–28	20,3	4,2
**ASBM**	12,3	13,186	9,39	10,78	6,878	7,5	11,74	9,8	15,54	13,72	8,07	7–16	10,8	2,8
**AAS**	45,9	42,102	47,8	38,55	33,39	39,8	49,95	41,4	50,5	44,44	47,28	33–51	43,7	5,3
**ASBa**	27,85	27,755	17,15	24,13	22,29	24,5	27,88	22,39	33,48	29,89	24,3	17–33	25,6	4,4
**PSE**	-	63,36	43,5	60,71	63,48	45	-	-	-	44,91	57,45	43–63	54,1	9,2
**ASE**	-	39,37	-	43,63	36,97	36,8	-	-	39,47	38,35	40,13	36–44	39,2	2,3
**A-P**	18,95	18,776	18,9	20,63	13,41	18,18	18,4	16,46	21,9	19,24	19,38	13–22	18,6	2,2
**Oc**	55,22	59,883	53,57	62,22	68,3	54,08	58,72	52,96	61,36	52,83	50,81	50–68	57,3	5,3
**MDS**	56,82	64,795	56,83	65,67	77,59	56,069	63,435	60,55	61,58	56,77	57,18	56–78	61,6	6,4
**PDS**	58,51	56,65	66,33	67,5	67,34	60,87	67,142	59,92	68,22	57,49	56,6	56–68	62,4	4,9
**GeI**	103,91	100,37	105,43	103,2	104,88	107,28	97,97	99,038	122,66	103,3	105,83	97–123	104,9	6,6
**Til**	133,4	132,48	122,97	126,3	127,08	126,37	152,69	128,05	150	136,9	135,99	122–153	133,8	9,7
**TaI(L)**	23,09	28,12	26,31	23,4	23,9	25,4	24,93	25,34	23,05	24,94	25,19	22–28	24,9	1,5
**TaI(H)**	130,23	136,9	131,9	136,9	132,96	133,62	154,93	131,56	151,3	134,53	134,39	130–155	137,2	8,2
**TiI/Gel**	1,2838	1,31992	1,16637	1,2238	1,2117	1,1779	1,55854	1,29294	1,22289	1,3253	1,2850	1–2	1,3	0,1
**GeII**	88,38	92,55	88,6	91,41	88,78	93,07	102,17	88,77	108,07	87,27	90,53	87–108	92,7	6,5
**TiII**	120,99	123,65	120,17	111,86	106,22	118,9	129,29	115,27	138,5	117,92	118,35	106–139	120,1	8,5
**TaII(L)**	25,17	23,83	32,53	23,96	24,87	32,36	37,92	22,66	28,9	24,51	24,83	22–38	27,4	4,9
**TaII(H)**	120,53	116,209	115,9	122,56	113,166	120,7	122,35	109,68	138,6	121,27	120,55	109–139	120,1	7,4
**TiII/GeII**	1,3690	1,3360	1,3563	1,2237	1,1964	1,2775	1,2654	1,299	1,282	1,3512	1,3073	0,1–1,3	1,3	0,1
**GeIII**	96,158	92,35	98,6	92,7	92,04	95,48	100,53	95,29	108,08	95,52	92,15	92–108	96,3	4,8
**TiIII**	173,1	179,207	172,71	166,82	154,75	165,2	187,69	116,58	201,5	172,43	168,48	116–202	169,0	21,3
**TaIII(L)**	18,8	21,879	28,63	20,97	22,4	20,21	24,85	21,07	19,61	19,3	23,06	19–29	21,9	2,9
**TaIII(H)**	124,5	116,464	127,29	127,57	114,97	124,8	125,4	115,49	135,25	134,53	118,8	114–135	124,1	7,1
**TiIII/GeIII**	1,800	1,94052	1,75162	1,800	1,68133	1,730	1,867	1,223	1,864	1,805	1,828	1–2	1,8	0,2
**AW/ISD**	1,4594	1,275605	1,522476	1,3729	1,365284	1,4816	1,607	1,377	1,659	1,4961	1,5011	1–2	1,5	0,1
**ISD/A-P**	3,807	1,921742	-	3,223	1,955640	3,880	-	3,991	4,230	3,637	3,486	1,9–4	3,3	0,8
**AW/A-P**	5,556	5,14013	5,75238	4,426	7,361	5,748	6,414	5,824	5,394	5,441	5,232	4–7	5,7	0,7
**St I**	25,27	38,71	28,78	29,52	41,32	30,23	24,42	35,03	35,67	29,54	28,25	23–41	31,5	5,4
**St II**	38,8	43,57	40,13	42,6	40,52	40,13	49,15	43,28	49,25	39,4	38,3	38–49	42,3	3,8
**CxI**	71,32	68,62	66,83	61,4	75,84	71	81,06	69,75	77,61	67,17	66,7	61–81	70,7	5,6
**CxII**	76,91	79,68	97,11	78,31	92,43	89	98,7	91,36	78,91	77,27	76,5	77–99	85,1	8,7
**CxIII**	85,82	88,49	83,1	85,35	88,14	87,53	97,37	69,9	87,9	84,04	75,34	70–97	84,8	7,2
**TiI/Aw**	1,267	1,37269	1,13107	1,383	1,287408	1,209	1,294	1,336	1,270	1,308	1,341	1,2–1,3	1,3	0,1
**TiIII/Aw**	1,149	1,28120	1,10532	1,225	1,56772	1,138	1,095	1,202	1,173	1,126	1,167	1–2	1,2	0,1
**AW/AL**	1,910	1,75529	2,16963	1,422	1,578	2,098	1,720	1,688	1,643	1,691	1,700	1,6–2,1	1,8	0,2
**AL/AAS**	1,201	1,30595	1,04833	1,665	1,87391	1,251	1,373	1,372	1,424	1,393	1,262	1,2–2	1,4	0,2

## 4 Discussion

Over 240 species belonging to the genus *Leptus* Latreille, 1796 have been documented in their larval stage, inhabiting various species; However, the larvae predominantly infest arthropods, with a preference for Araneae, Coleoptera, Diptera, Hemiptera, Lepidoptera, Opiliones, and Orthoptera [33]. The majority of the reports rely on descriptions of the larvae [34], and just four species are described by the NCBI: *Leptus ignotus*, *Leptus tridentatus*, *Leptus oudemansi and Leptus sidorchukae* [35]. More than 54 species of *Leptus* have been described in the America continent (North, Central and South), at least 32 species for South America [28,30,32,36,37] (Table 3). Here, we report the first record in Colombia of a mite species closely related to *L*. *alberti* n.spp., which is part of the Trombidiformes order, Prostigmata suborder, anystina infraorder. For the *Leptus* (L), there are two previous additional reports in Colombia. The first report of *Leptus olafi* in Colombia, Panamá and Venezuela was in 1991 [32]; in addition to *Leptus stefani* in Colombia [32,38].

**Table 3:** Species of *Leptus* Latreille, 1796 documented in South America.

	Species	Host	Country	Ref
1	*L*. *adaminae* (Haitlinger, 2004) [39]	Plants	Brazil	[30,39]
2	*L*. (Amaroptus) *vuki* Haitlinger, 2000 [40]	Orthoptera	Perú	[40–42]
3	*L*. (*L*.) *alberti* Haitlinger, 1991	*Homophoeta personata* (Illiger, 1807), (Coleoptera: Chrysomelidae: Halticinae)	Brazil	[32]
4	*L*. (*L*.) *annikae* Haitlinger, 2000 [40]	Orthoptera	Perú	[40,41]
5	*L*. (*L*.) *ariel (*Southcott, 1992) [27]	*A*. *mellifera* Linnaeus, 1758 (Hymenoptera: Apidae), Eumolpinae (Coleoptera: Chrysomelidae)	Guatemala, Perú	[42,43]
6	*L*. (*L*.) *brasilicus* sp.nov (Haitlinger, Sundic & amp; Pompermaier, 2017) [44]	pitfall traps, free living on the ground	Brazil	[44]
7	*L*. (*L*.) *cabareticus* (Haitlinger, 2004) [39]	Herbaceous plants	Dominican Republic	[29,45]
8	*L*. *candangus* (Sundic, Haitlinger and Pompermaier, 2017) [44]	pitfall traps	Brazil	[46]
9	*L*. *cyrili* (Haitlinger, 1991)	Lycidae (Coleoptera)	Brazil	[47]
10	*L*. *fozicus* (Haitlinger, 2004) [39]	plants	Brazil	[46,48]
11	*L*. (*L*.) *filipinae* Haitlinger, 2000 [40]	Lampyridae (Coleoptera), Hemiptera undet.	Costa Rica, México	[40–42]
12	*L*. *gagzoi* (Oudemans, 1910) [49]	Tettigonidae (Orthoptera)	(Panama, Trinida and Tobago)	[38,46,49]
13	*L*. (*L*.) *guarani* n. sp.	*Acanthogonyleptes editus* (Rewer, 1943) (Opilones: Gonyleptidae)	Brazil	[42]
14	*Leptus* (*L*.) *haitlingeri*	Fidena (Fidena) sp. (Diptera: Tabanidae)	Brazil	[28,42]
15	*L*. (*L*.) *hringuri* (Haitlinger, 2000) [40]	Brachyceridae (Bracchycerinae) (Coleoptera)	Perú	[40,46]
16	*L*. *(L) iguacuicus* (Haitlinger, 2004) [39]	plants	Brazil	[44,46]
17	*Leptus* (*L*.) *lomani* (Oudemans, 1902) [50]	Discocyrtus funestus (Opiliones: Gonyleptidae)	Chile	[50]
18	*L*. (*L*.) *maldonadoicus* (Haitlinger, 2000) [40]	Orthoptera	Perú	[40,46]
19	*L*. (*L*.) *mariani* (Haitlinger, 1991)	*Stolas festiva* (Klug, 1829) (Coleoptera: Chrysomelidae: Cassidinae)	Brazil	[47]
20	*L*. (*L*.) *multisolenidiae* Mayoral and Barranco, 2011 [51]	Episomacris gruneri (Descamps and Amédégnato, 1970) (Orthoptera: Acrididae)	French Guiana	[51]
21	*L*. (*L*.) *nikanori* Haitlinger, 2000 [40]	*Anaulacomra* sp. (Tettigonidae), *Pseudoproscopia scabra* (Klug, 1820) (Proscopidae) (Orthoptera), *Gonyleptes gonyleptoides* H.E.M. Soares and B. A. Soares, 1945) (Opiliones: Gonyleptidae)	Costa Rica, French Guaina	[40,42,51]
22	*L*. (*L*.) *olafi* (Haitlinger, 1991)	Cicindellidae (Coleoptera), Orthoptera	Colombia, Venezuela, Chile,	[38]
23	*L*. *onnae* (Haitlinger, 2000) [40]	Plants	Brazil, México	[29,39,48,52]
24	*L*. (*L*.) *oudemansi* (Karpinen, 1958) = *L*. (*L*). *gracilipes* (Oudemans, 1910) [49]	*Cynorta* sp. (Opiliones: Cosmetidae), arachnids (Opiliones) and Coleoptera (Cleridae)	Surinam	[49]
25	*L*. (*L*.) *planaltensis* Haitlinger, Šundić & Pompermaier, 2017. [44]	pitfall traps	Brazil	[44,46]
26	*L*. (*L*.) *schedingi* (Oudemans, 1911)	lepidoptera	Chile	[46]
27	*L*. (*L*.) *sieversi* (Oudemans, 1911)	arthropods	Venezuela	[30,38]
28	*L*. (*L*.) *simonettae* Haitlinger, 2000 [40]	Acrididae (Orthoptera)	Guatemala, Honduras Brazil	[40]
29	*L*. (*L*.) *stefani* Haitlinger, 1991	Ptychoderes sp. (Coleoptera: Anthribiidae)	Colombia,	[36,47]
30	*L*. *stieglymayri* (Oudemans, 1905)	arachnids (Opiliones) and Coleoptera (Cleridae)	Brazil	[42,46,50]
31	*L*. (*L*.) *stolae* (Haitlinger, 1987) [53]	*Stolas aenea* (Olivier, 1790); S. *chalybaea* (Germar, 1824); *S*. *nudicollis* (Boheman, 1850); *S*. *oblita* (Boheman, 1850) (Coleoptera: Chrysomelidae: Cassidinae)	Brazil	[42,53]
32	*L*. (*L*.) *tiranicus* Haitlinger, 2006 [38]	Orthoptera	Venezuela	[38]
33	*L*. (*L*.) *ursyni* (Haitlinger, 1991)	*Scotobius planatus* Erichson, 1834 (Coleoptera: Tenebrionidae)	Chile	[46,47]

The presence of *Leptus* in wild *A*. *mellifera* and *P*. *peckolti*, *P*. *eutaeniata* and *T*. *angustula* is relevant considering that this mite may be detected in the gut and muscle-tissue of *Leptus* (*Leptus sayi*, 15,4% prevalence; *Leptus lomani*, 14.3% prevalence*)* [21]. Future studies are required to identify whether *L*. *alberti* is infected with spiroplasmas and the effect or role on host interaction (female and males) in other populations.

Although there have been previous reports of *L*. *olafi* and *L*. *stefani* in Colombia, this is the first documentation of a new species *L*. *alberti* n.sp parasitizing bees in Colombia. In parallel, *Leptus* spp., has been observed parasitizing stingless bees (Apidae:Meliponini) in Argentina [20]. *A*. *mellifera* Africanized honey bees have also been detected with *Leptus* spp in 26 (87%) of 30 study colonies. [23]. Flechtman et al. (1980) [56] reported in Lima, Peru, fourteen *Leptus* sp. larvae that were collected from honeybees, *A*. *mellifera* L. These larvae were attached to the intersegmental membranes of abdominal tergites and sternites. This is an old association that comes from the Eocene, as tests from ambar fossils suggest **[54–56].**

Different factors potentially influence the association between *L*. *alberti* and *P*. *peckolti*, including the nesting habitat, characterized by locations in holes within walls or proximity to termite colonies. These habitats are typically situated in tropical forests and anthropogenic areas along the Pacific coast and Andean valleys, spanning elevations between 10 and 2,850 meters above sea level [13]. The nesting preferences of *P*. *peckolti* possibly expose them to a higher risk of encountering these parasites, which could contribute to the observed findings.

The identification of *Leptus* spp. in free-living colonies of *A*. *mellifera*, *P*. *peckolti*, *P*. *eutaeniata*, and *T*. *angustula* in the wild is likely attributed to the limited human intervention in these ecosystems, facilitating the discernment of these bee populations. The absence of *Leptus* spp. in regions where *A*. *mellifera* is actively managed by beekeepers implies a discernible pattern. Investigations are imperative to elucidate the specific role of *Leptus* spp. as potential vectors of diseases in free-living colonies, thereby contributing to a more nuanced understanding of the dynamics governing these interactions.

Comparative morphology remains a constant approach to species identification [57–59]; However, *Leptus* species in South America need to be redescribed since the currently available information is insufficient. New drawings and meristic data are also needed.

There is a low number of examined specimens and a high dependence on metric data to separate species. In the case of *L*. *alberti*, there are metric data of limited value. This classification uses mostly non-metric characters of published descriptions for differentiation of *L*. *alberti* from other possible species. Cryptic species are phylogenetically closely related that exhibit no unambiguous morphological differences. In order to readily allow their distinction, an integrative taxonomic approach with additional evidence (Behaviour, physiology, ecology) is required, in addition to DNA barcoding methods that help to understand these findings.

## 5 Conclusions

This study represents the primary complete morphometric documentation of a mite *L*. *alberti* n. spp. in free-living colonies of *A*. *mellifera*, as well as in other free-living species such as *P*. *peckolti*, *P*. *eutaeniata*, and *T*. *angustula* within the Cauca and Valle del Cauca Departments. This discovery contributes novel insights to the existing understanding of *Leptus* species in South America, thereby enriching the catalog of documented species in the region. Furthermore, the study underscores the imperative for future investigations aimed at ascertaining whether *L*. *alberti* is infected with Spiroplasmas and delving into the potential effects or roles in host interactions. Given the dynamic nature of arthropod associations, it is crucial to explore and elucidate the multifaceted roles and impacts of these interactions in diverse ecosystems. Future studies require an integrative taxonomic approach with additional evidence.

## Supporting information

S1 Fig *Leptus alberti* n. sp. Comparison Cauca and Valle populations. (PDF)
