# Supplementary material for: Leptus alberti n. sp. (Trombidiformes: Erythraeidae) parasitizing free-living colonies of Apis mellifera, Partamona peckolti, Paratrigona eutaeniata and Tetragonisca angustula in Totoró and Valle del Cauca, Colombia
Source: PLoS One. 2024 Dec 16;19(12):e0311409. doi: 10.1371/journal.pone.0311409 (PMC11649092; doi:10.1371/journal.pone.0311409)
Supplement: S1 Fig — (PDF) [file pone.0311409.s001.pdf]

**_Leptus alberti_ n. sp. (Trombidiformes: Erythraeidae) parasitizing free-living colonies of _Apis mellifera_, _Partamona peckolti_, _Paratrigona eutaeniata_ and _Tetragonisca angustula_ in Totoró and Valle del Cauca, Colombia**

Brayan Alexander Sanchez-Quilindo[1], Harby Leandro Pizo-Barona [1], Javier Antonio Benavides-Montaño[1]

1.Department of Animal Science. Universidad Nacional de Colombia, Sede Palmira, Carrera 32 # 12-00, Valle del Cauca, Colombia

Correspondence: abenavidesm@unal.edu.co

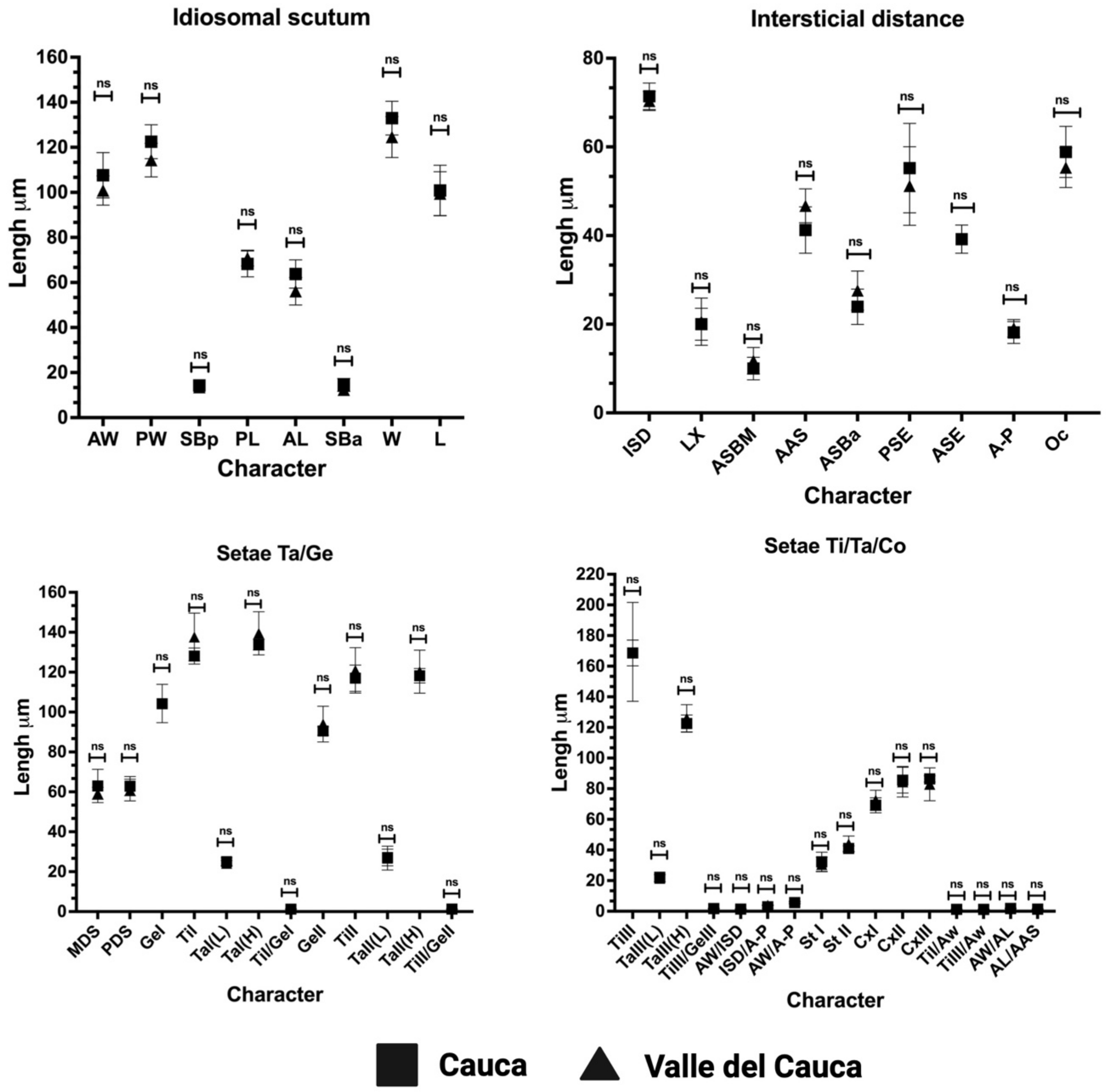


**Figure S1**. ***Leptus alberti* n. sp. Comparison Cauca and Valle populatons.** The statistical analysis of all data was conducted using GraphPad Prism 10 software. For the measures comparisons from *Leptus alberti* n. sp., a one-way analysis of variance (ANOVA) was performed, followed by a post hoc Bonferroni's multiple-comparison test. No significant difference were found.
